# Male Sexual Dysfunction and the Perpetration of Intimate Partner Violence

**DOI:** 10.1177/10778012231174348

**Published:** 2023-05-21

**Authors:** Terrence D. Hill, Ginny Garcia-Alexander, Katelyn Sileo, Chantal Fahmy, Alexander Testa, Rebecca Luttinen, Ryan Schroeder

**Affiliations:** 1 Department of Sociology, One UTSA Circle, 12346 University of Texas at San Antonio, San Antonio, TX, USA; 2 Department of Sociology, 12346 University of Texas at San Antonio, San Antonio, TX, USA; 3 Department of Public Health, 12346 University of Texas at San Antonio, San Antonio, TX, USA; 4 Department of Criminology & Criminal Justice, 12346 University of Texas at San Antonio, San Antonio, TX, USA; 5 Department of Management, Policy and Community Health, 12340 University of Texas Health Science Center at Houston, San Antonio, TX, USA; 6 Department of Demography, 12346 University of Texas at San Antonio, San Antonio, TX, USA; 7 Department of Criminal Justice and Criminology, 7604 Georgia Southern University, Statesboro, GA, USA

**Keywords:** sexual dysfunction, erectile dysfunction, masculinity, anger, intimate partner violence

## Abstract

We contribute to our understanding of the social epidemiology of intimate partner violence (IPV) by developing a mediation model that frames IPV as an outcome of male sexual dysfunction (performance anxiety and erectile dysfunction) and the mechanisms of masculine discrepancy stress (the perceived failure to conform to internalized normative expectations of masculinity) and anger. Our mediation analyses of recently collected data from the 2021 *Crime, Health, and Politics Survey* (CHAPS), a national probability sample of 792 men, confirmed that sexual dysfunction was indirectly associated with the perpetration of any IPV, physical IPV, and sexual IPV through the compound path of masculine discrepancy stress and anger.

## Introduction

Are men who experience sexual dysfunction (e.g., performance anxiety and erectile dysfunction) more likely to commit acts of intimate partner violence (IPV) than other men? Although research has consistently shown that sexual dysfunction can be an outcome among women experiencing IPV (Campbell, 2002; Coker, 2007), few studies have considered whether sexual dysfunction might also lead some men to perpetrate IPV. While there is at least some evidence to suggest that small criminal and clinical samples of sexual offenders often present with symptoms of sexual dysfunction (Bownes, 1993; Dwyer & Amberson, 1989; Jones et al., 2010), male college students who admit to engaging in “sexual aggression against women” may also exhibit higher rates of “erectile and orgasmic difficulties, and more sexual inhibition due to the threat of performance failure” (Carvalho et al., 2013, p. 1744). In this article, we extend our understanding of the social epidemiology of IPV by developing and testing a mediation model that integrates theories from criminology and gender studies to frame IPV as an outcome of male sexual dysfunction and the mechanisms of masculine discrepancy stress (the perceived failure to conform to internalized normative expectations of masculinity) and anger.

## Theoretical Model

**Figure 1.**
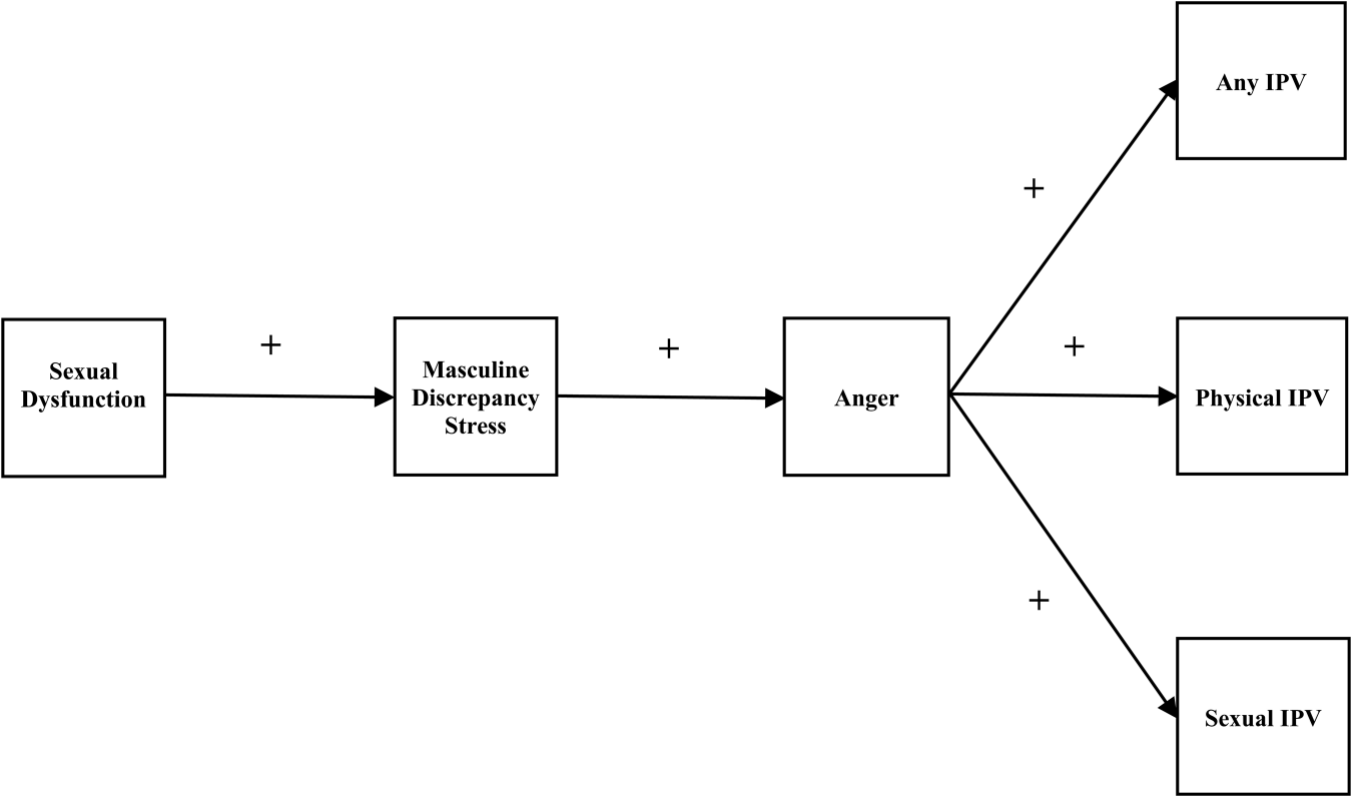
Theoretical Model Linking Male Sexual Dysfunction and Intimate Partner Violence.

Our theoretical model proposes that male sexual dysfunction increases the risk of IPV perpetration through the mechanisms of masculine discrepancy stress and anger (see Figure 1). Our model is informed by the integration of theoretical frameworks from criminology (general strain theory [GST]) and gender studies (compensatory masculinity). In this section, we describe the relevance of these frameworks and explore the theoretical and empirical basis for each link in our proposed theoretical model.

### General Strain Theory

GST argues that people engage in criminal behavior because they experience various strains which generate a range of negative emotions or affective states (Agnew, 1992, 2001, 2007). Strains include the failure to achieve positively valued goals (e.g., monetary success), the presence of noxious or negatively valued stimuli (e.g., living in a dangerous neighborhood), and the removal of positively-valued possessions or relationships (e.g., the loss of a loving parent). Negative emotions range from frustration and anger to anxiety and depression. GST frames criminal behavior as an illegitimate corrective action or means of addressing strains or coping with negative affective states. When our model is interpreted through the lens of GST, sexual dysfunction (e.g., the inability to achieve or maintain an erection during sexual activity) and masculine discrepancy stress are considered strains because they represent a failure to achieve the positively valued goals of health, virility, and masculinity among men (Agnew, 2007; Messerschmidt, 1993; Schroeder et al., 2011). To the extent that these strains are sufficient to elicit feelings of anger in men, any subsequent acts of IPV become a criminal means of coping. Empirical tests of GST have consistently attributed behaviors related to aggression and violence to feelings of anger and hostility (De Coster & Cornell Zito, 2010; Ganem, 2010; Jang & Johnson, 2003; Mazerolle et al., 2000, 2003).

### Compensatory Masculinity

Compensatory masculinity is a process by which men attempt to offset threats to their manhood by enhancing previous behavioral expressions of masculinity or by projecting masculinity in new ways (Courtenay, 2000; Hill et al., 2021; Melzer, 2002; Pyke, 1996). The concept of compensatory masculinity has been used to explain the dominant behavior of men relative to women in general and to lower status men (e.g., straight men vs. gay/bisexual men). When applied to our model, compensatory masculinity helps us to understand the precise motivations of men. GST tells us that men are motivated to alleviate strain and negative affective states. Compensatory masculinity tells us that men are driven to demonstrate power and status in a relational context. In fact, several studies frame IPV as a primary tactic of compensatory masculinity (Berke et al., 2017; Cheung & Chiu, 2021; DeMaris et al., 2003; Johnson & Ferraro, 2000; Melzer, 2002; Pyke, 1996; Reidy et al., 2014, 2018).

### Sexual Dysfunction and Masculine Discrepancy Stress

The first link in our model suggests that men experiencing sexual dysfunction will tend to report higher levels of masculine discrepancy stress. Although fluid and context specific, the masculine role tends to be defined by independence, aggressiveness, risk-taking, virility, stoicism, and sexual prowess (Connell, 1987; Courtenay, 2000; Hill & Needham, 2013; Sileo & Kershaw, 2020). Sexual dysfunction is likely to pose a direct challenge to this role (Fergus et al., 2002; Oliffe, 2005; Potts, 2000). Oliffe (2005, p. 2250) explains that “penis-centered sexuality can create confusion between personhood or identity and one's sexual organ, and when the penis does not operate according to dominant ideals, men equate this with a loss of manhood.” According to Potts (2000, p. 90), “the absence of—or difficulty in ‘achieving’ and ‘maintaining’— a robust ‘hard on’ in appropriate circumstances presents as a disastrous affliction in the male—an abnormality, a failure to stand up and be counted as a ‘real’ man.”

### Masculine Discrepancy Stress and Anger

The second link in our model suggests that masculine discrepancy stress will be positively associated with feelings of anger. When boys and men fail to meet internalized normative masculine ideals, they are often subjected to the injustice of social sanctions that range from disapproval and rejection to bullying and victimization (Parrott, 2009; Reidy et al., 2018; Reisner et al., 2015). Studies also indicate that threats to masculinity can be emotionally distressing for men because they represent the loss of identity, status, and power (Berke et al., 2017; Chambers et al., 2017; Dahl et al., 2015; Eisler et al., 1988; Sileo et al., 2022; Vescio et al., 2021). Anger is generally contextualized in response to “status injury” and “the loss of social recognition” (Lyman, 2004). Moreover, Dahl et al. (2015, p. 244) note that “anger is a critical emotional response to masculinity threats” because “people feel angry when prevented from realizing a goal, or a desired end-state, and masculinity is a desired end-state that men work hard to achieve and maintain.”

### Anger and IPV

The final link in our model suggests that anger will be positively associated with the perpetration of IPV. It is well known that when people are “consciously aware of feeling angry,” they can be “physically mobilized by anger” (Lyman, 2004, p. 136). While men use anger to project power and strength (Brody, 1999), Dahl and colleagues (2015, p. 244) explain that, in the context of threats to masculinity, “anger is an emotion that motivates corrective action to remove, recover from, or appease a threat.” In the manner of compensatory masculinity, acts of IPV become “intentional tactics to control through displaying, reinforcing, or reclaiming masculinity” (Cheung & Chiu, 2021, p. 79). Over the past two decades, numerous studies have confirmed associations between emotions like anger and hostility and IPV (Birkley & Eckhardt, 2015; Norlander & Eckhardt, 2005; Sileo et al., 2022).

## Data

For this investigation, we use data from the 2021 *Crime, Health, and Politics Survey* (CHAPS). CHAPS is based on a national probability sample of 1,771 community-dwelling adults aged 18 and over living in the United States. Respondents were sampled from the National Opinion Research Center's (NORC) *AmeriSpeak*© panel, which is representative of households from all 50 states and the District of Columbia (https://amerispeak.norc.org/Documents/Research/AmeriSpeak%20Technical%20Overview%202019%2002%2018.pdf). Sampled respondents were invited to complete the online survey in English between May 10, 2021 and June 1, 2021. The data collection process yielded a survey completion rate of 30.7% and a weighted cumulative response rate of 4.4%. The weighted cumulative response rate is the overall survey response rate that accounts for survey outcomes in all response stages, including the panel recruitment rate, panel retention rate, and survey completion rate. It is weighted to account for the sample design and differential inclusion probabilities of sample members. Our cumulative response rate is within the typical range of high-quality general population surveys (4–5%).

The multistage probability sample resulted in a margin of error of ±3.23% and an average design effect of 1.92. Margin of error is defined as half the width of the 95% confidence interval (CI) for a proportion estimate of 50% adjusted for design effect. A figure of ±3.23% is therefore the largest margin of error possible for all estimated percentages based on the study sample. A margin of error of plus or minus 3.23 percentage points at the 95% confidence level means that if we fielded the same survey 100 times, we would expect the result to be within 3.23 percentage points of the true population value 95 of those times. A margin of error of 3.00 is considered very good. The average design effect is the variance under the complex design divided by the variance under a simple random sampling design of the same sample size. The design effect is variable-specific, and the reported value is the average design effect calculated for a set of key survey variables. Design effects account for deviations from simple random sampling with a 100% response rate. A design effect of 1.92 is very good because it means that the variance is only about twice as large as would be expected with simple random sampling. The median self-administered web-based survey lasted approximately 25 min. All respondents were offered the cash equivalent of $8.00 for completing the survey. The survey was reviewed and approved by the Institutional Review Boards at NORC and the lead author's university. Informed consent was obtained from all participants. The primary purpose of CHAPS is to document the social causes and social consequences of various indicators of health and well-being in the United States during the coronavirus (COVID-19) pandemic. The omnibus survey includes measures of psychosocial characteristics, religious beliefs and experiences, political views and behaviors, neighborhood conditions, experiences with crime and police, stressful life events, health behavior and health lifestyles, mental health, physical health, sexual and reproductive health, and sociodemographic characteristics.

## Measures

### Intimate Partner Violence

We measure IPV with six items from the Short Form of the Revised Conflict Tactics Scales (Straus & Douglas, 2004). Four items assessed the perpetration of physical assault and injury: (a) “Have you ever pushed, shoved, or slapped a partner?” (b) “Have you ever punched, kicked, or beat-up a partner?” (c) “Has a partner ever had a sprain, bruise, small cut, or felt pain the next day because of a fight with you?” (d) “Has a partner ever needed professional medical treatment because of a fight with you?” Two additional items assessed the perpetration of sexual coercion: (e) “Have you ever insisted on having sex with a partner without a condom?” (f) “Have you ever used force (like hitting, holding down, or using a weapon) to make a partner have sex?” Original response categories included (a) yes, in the past year, (b) yes, but not in the past year, and (c) no, this has never happened. To account for limited rates of occurrence, all IPV items were dichotomized. Our measure of any IPV distinguishes between (1) men who have perpetrated any act of physical or sexual violence in their lifetime and (0) men who have not (Items a–f). Our measure of physical IPV distinguishes between (1) men who have perpetrated any act of physical assault or injury in their lifetime and (0) men who have not (Items a–d). Finally, our measure of sexual IPV distinguishes between (1) men who have perpetrated sexual violence in their lifetime and (0) men who have not (Items e and f).

### Sexual Dysfunction

Sexual dysfunction is measured as the mean response to three items: (a) whether a respondent felt “anxious about their ability to perform sexually” in the past year (1 =  *never* to 5  =  *always*), (b) whether a respondent had “trouble achieving or maintaining an erection during sexual activity” in the past year (1  =  *never* to 5  =  *always*), and (c) whether a doctor or health professional had ever prescribed a respondent “any medication for erectile dysfunction or ED (e.g., Viagra, Levitra, or Cialis)” (1  =  *yes*; 0  =  *no*). The first two items were drawn from the work of Laumann et al. (1992). The medication question was drawn from the Erectile Dysfunction Questionnaire developed by the Brady Urological Institute at Johns Hopkins Medicine. To account for metric differences, each sexual dysfunction item was standardized before indexing. An exploratory principal components analysis with varimax rotation produced a single component (eigenvalue  =  1.97), with loadings ranging from 0.69 to 0.88. A reliability analysis also suggested adequate internal consistency for three items (α  =  0.73).

### Masculine Discrepancy Stress

Masculine discrepancy stress is measured as the mean response to four items (Reidy et al., 2014). Respondents were asked to indicate their level of agreement with the following statements: (a) “I wish I was more ‘manly.’” (b) “Sometimes I worry about my masculinity.” (c) “I worry that people judge me because I am not like the typical man.” (d) “I worry that others find me less attractive because I’m not as ‘manly’ as other guys.” Response categories for these items ranged from (1) *strongly disagree* to (5) *strongly agree*. An exploratory principal components analysis with varimax rotation produced a single component (eigenvalue  =  3.15), with loadings ranging from 0.85 to 0.91. A reliability analysis also suggested excellent internal consistency (α  =  0.91).

### Anger

Anger is measured as the mean response to three items drawn from the How I Feel Instrument (Petersen & Kellam, 1977). Respondents were asked to indicate how often in the past 30 days they (a) felt angry, (b) lost their temper, and (c) yelled at people. Response categories for these items ranged from (1) *never* to (5) *always*. An exploratory principal components analysis with varimax rotation produced a single component (eigenvalue  =  2.36), with loadings ranging from 0.86 to 0.93. A reliability analysis also suggested good internal consistency (α  =  0.86).

### Background Variables

All analyses include several potential background correlates of our focal variables, including *age* (continuous years), *race/ethnicity* (dummy variables for non-Hispanic whites, non-Hispanic black, Latino, and other races/ethnicities); *college degree* (1  =  *four-year college degree or higher*; 0  =  *otherwise*), *employment* (1  =  *employed full- or part-time*; 0  =  *otherwise*), *annual household income* (1  =  <$10,000 to 9  =  ≥$150,000), *relationship status* (dummy variables for married, living with a partner, and no partner), and *financial strain* (a mean index of items assessing the extent to which a respondent's household had trouble paying for health care, monthly bills, and food; α  =  0.89).

## Analysis

Given our focus, the analytic sample is limited to a national probability sample of 844 men (total possible sample size). Due to listwise deletion of missing data on anger (*n*  =  3), any IPV (*n*  =  13), physical IPV (*n*  =  13), sexual IPV (*n*  =  15), masculine discrepancy stress (*n*  =  31), and sexual dysfunction (*n*  =  41), our analytic sample was reduced to 792 respondents or 94% of the total possible sample of men.

Post-stratification weights were used in supplemental analyses to assess sampling error and nonresponse bias. NORC developed post-stratification weights for CHAPS via iterative proportional fitting or raking to general population parameters derived from the *Current Population Survey* (https://www.census.gov/programs-surveys/cps/data.html). These parameters included age, sex, race/ethnicity, education, and several interactions (Age × Sex, Age × Race, and Sex × Race).

**Figure 2.**
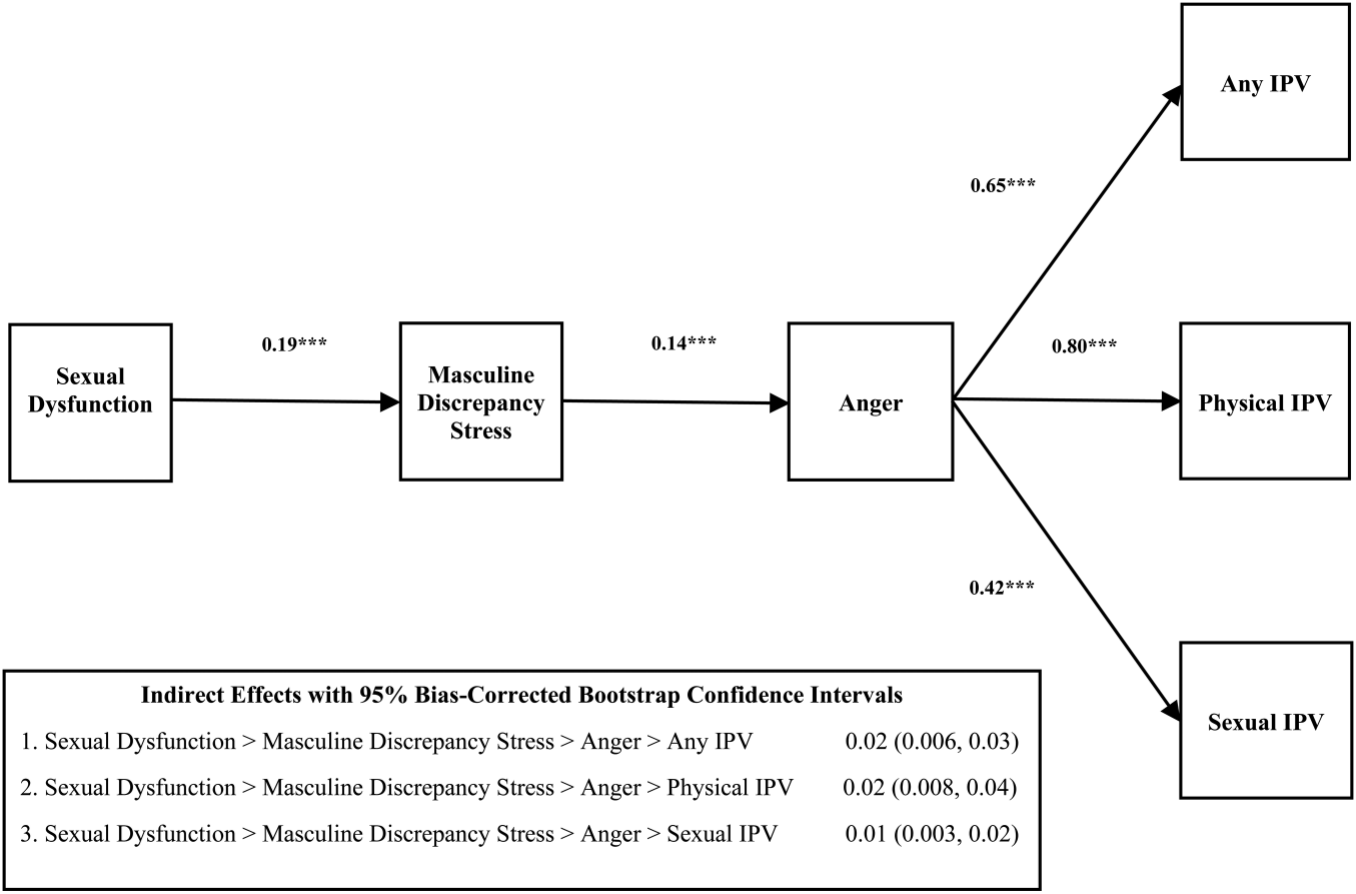
Empirical Model Linking Male Sexual Dysfunction and Intimate Partner Violence.

Table 1 presents descriptive statistics for all study variables, including variable ranges, sample means, and standard deviations. In Table 2, we use ordinary least squares (OLS) regression to model our continuous outcomes (masculine discrepancy stress and anger) and binary logistic regression to model our dichotomous outcomes (any IPV, physical IPV, and sexual IPV). All regression models present unstandardized coefficients and two-tailed statistical tests. We use conditional process analysis to formally assess our serial multiple mediator model (Hayes, 2018). Figure 2 shows our empirical model with unstandardized indirect effects (IEs) and 95% bootstrap CIs obtained from 20,000 bootstrap samples. Bootstrap CIs are preferable to normal theory-based mediation tests because IEs (products of component paths) are not normally distributed (Hayes, 2018).

**Table 1.** Descriptive Statistics.

	Range	Mean	Standard deviation
Any IPV	0 to 1	0.32	
Physical IPV	0 to 1	0.16	
Sexual IPV	0 to 1	0.22	
Sexual dysfunction	−0.81 to 2.26	−0.002	0.81
Anger	1 to 5	2.15	0.83
Masculine discrepancy stress	1 to 5	2.01	0.88
Age	18 to 91	49.11	17.42
White	0 to 1	0.67	
Black	0 to 1	0.09	
Latino	0 to 1	0.17	
Other race/ethnicity	0 to 1	0.07	
College degree	0 to 1	0.34	
Employed	0 to 1	0.65	
Household income	1 to 9	5.67	2.21
Financial strain	1 to 5	1.60	0.91
Married	0 to 1	0.57	
Partnered	0 to 1	0.06	
No partner	0 to 1	0.37	

*Note*. *n*  =  792. IPV = intimate partner violence.

**Table 2.** Ordinary Least Squares Regression of Masculinity Discrepancy Stress and Binary Logistic Regression of IPV.

	Masculine discrepancy stress	Anger	Any IPV	Physical IPV	Sexual IPV
Sexual dysfunction	0.19***	0.19***	0.25*	0.25	0.26*
Masculine discrepancy stress		0.14***	−0.11	0.07	−0.17
Anger			0.65***	0.80***	0.42***
Age	−0.01***	−0.01***	0.003	0.02*	−0.01
Black	−0.20	0.15	0.75**	1.34***	0.48
Latino	0.05	−0.14	0.52*	0.41	0.36
Other race/ethnicity	0.02	0.11	−0.12	−0.40	0.21
College degree	0.09	0.12*	−0.11	0.32	−0.39
Employed	−0.08	−0.02	0.30	0.15	0.33
Household income	−0.05***	−0.002	−0.05	0.01	−0.08
Financial strain	0.07	0.20***	0.005	0.24*	−0.08
Married	−0.05	0.10	0.22	0.34	0.22
Partnered	0.04	0.28*	0.56	1.18**	0.003

*Note*. *n*  =  792. IPV = intimate partner violence. Shown are unstandardized ordinary least squares (masculine discrepancy stress and anger) and binary logistic (IPV) regression coefficients with two-tailed tests.

*p < 0.05. **p < 0.01. ***p < 0.001.

## Results

### Regression of Masculine Discrepancy Stress

The first column of Table 2 indicates that sexual dysfunction is positively associated with masculine discrepancy stress (*b*  =  0.19, *p* < 0.001). In other words, men who report more problems with performance anxiety and erectile dysfunction also tend to report more concerns about conforming to masculine gender roles. Men who are older (*b*  =  −0.01, *p* < 0.001) and report higher household incomes (*b*  =  −0.05, *p* < 0.001) also tend to exhibit lower levels of masculine discrepancy stress. Standardized coefficients (not shown) indicate that the magnitude of the association with masculine discrepancy stress is comparable for age (β  =  −0.19) and sexual dysfunction (β  =  0.18). The association between household income and masculine discrepancy stress is noticeably weaker (β  =  −0.13).

### Regression of Anger

The second column of Table 2 shows that sexual dysfunction (*b*  =  0.19, *p* < 0.001) and masculine discrepancy stress (*b*  =  0.14, *p* < 0.001) are positively associated with anger. These patterns suggest that men who report more problems with performance anxiety/erectile dysfunction and more concerns about conforming to masculine gender roles also tend to exhibit more anger-related emotions and behaviors like feeling angry and losing one's temper. Younger men (*b*  =  −0.01, *p* < 0.001), men with college degrees (*b*  =  0.12, *p* < 0.05), and men experiencing financial difficulties (*b*  =  0.20, *p* < 0.001) tend to exhibit higher levels of anger. Unmarried men who live with a partner also tend to be angrier than men without partners (*b*  =  −0.28, *p* < 0.05). The strongest associations with anger are observed for financial strain (β  =  0.21), sexual dysfunction (β  =  0.19), age (β  =  −0.17), and masculine discrepancy stress (β  =  0.15). Associations with being partnered (β  =  0.08) and having a college degree (β  =  0.07) are less pronounced.

### Regression of IPV

The last three columns of Table 2 reveal that sexual dysfunction increases the log odds of having perpetrated any IPV (*b*  =  0.25, *p* < 0.05) and sexual IPV (*b*  =  0.26, *p* < 0.05), but not physical IPV (*b*  =  0.25, *p* > 0.05). These unstandardized logistic regression coefficients can be converted to odds ratios (ORs) through exponentiation (*e*^b^) and further manipulated ([OR − 1]  ×  100) to describe the percentage difference in the odds of having perpetrated any IPV for each one-unit change in the independent variable of interest. Accordingly, each unit increase in sexual dysfunction increases the odds of having perpetrated any IPV by 28% ([1.28 − 1]  ×  100) and sexual IPV by 30%. The odds of having perpetrated physical IPV were comparable across levels of sexual dysfunction. Taken together, these results suggest that sexual dysfunction is more strongly associated with sexual IPV than physical IPV. Although masculine discrepancy stress is unrelated to any IPV (*b*  =  −0.11, *p* > 0.05), physical IPV (*b*  =  0.07, *p* > 0.05), and sexual IPV (*b*  =  −0.17, *p* > 0.05), anger is positively related to any IPV (*b*  =  0.65, *p* < 0.001), physical IPV (*b*  =  0.80, *p* < 0.001), and sexual IPV (*b*  =  0.42, *p* < 0.001). In other words, each unit increase in anger increases the odds of having perpetrated any IPV by 92%, physical IPV by 122%, and sexual IPV by 52%.

There are also several associations with background variables. The log odds of having perpetrated any IPV are higher for non-Hispanic black men (*b*  =  0.75, *p* > 0.01) and Latino men (*b*  =  0.52, *p* > 0.05) than for non-Hispanic white men. The log odds of physical IPV perpetration are also higher for older men (*b*  =  0.02, *p* > 0.05), non-Hispanic black men (*b* =  1.34, *p* > 0.001), men experiencing financial strain (*b*  =  0.24, *p* > 0.05), and unmarried partnered men (*b*  =  1.18, *p* > 0.01).

The strongest associations with IPV perpetration are observed for anger (β  =  0.27), sexual dysfunction (β  =  0.11), non-Hispanic black (β  =  0.11), and Latino (β  =  0.10). The strongest associations with physical IPV perpetration include anger (β  =  0.31), age (β  =  0.18), non-Hispanic black (β  =  0.18), partnered (β  =  0.13), and financial strain (β  =  0.10). Finally, anger (β  =  0.18) and sexual dysfunction (β  =  0.11) exhibit the strongest associations with sexual IPV perpetration. Across outcomes, anger is the strongest and most consistent risk factor for IPV perpetration. This is understandable because anger is the most proximal link to violent behavior in our proposed model. Although IPV associations with sexual dysfunction and non-Hispanic black race are less pronounced, they are relatively consistent across multiple outcomes.

### Mediation Analyses

Figure 2 shows our empirical model with unstandardized IEs and 95% bootstrap CIs. The results of our mediation analyses suggest that the serial IE of sexual dysfunction through masculine discrepancy stress *and* anger is different from zero (because none of the CIs contain zero) for any IPV (IE  =  0.02, 95% CI  =  0.006, 0.03), physical IPV (IE  =  0.02, 95% CI  =  0.008, 0.04), and sexual IPV (IE  =  0.01, 95% CI  =  0.003, 0.02). Although we are primarily interested in our focal serial multiple mediator model, we did observe that the simple IE of sexual dysfunction through anger alone is different from zero for any IPV (IE  =  0.12, 95% CI  =  0.07, 0.19), physical IPV (IE  =  0.15, 95% CI  =  0.08, 0.24), and sexual IPV (IE  =  0.08, 95% CI  =  0.03, 0.14). However, the simple IE of sexual dysfunction through masculine discrepancy stress alone is null (not different from zero) for any IPV (IE  =  −0.02, 95% CI  =  −0.06, 0.15), physical IPV (IE  =  0.01, 95% CI  =  −0.03, 0.06), and sexual IPV (IE = −0.03, 95% CI  =  −0.08, 0.01). To summarize, we confirmed that sexual dysfunction is at least indirectly associated with IPV through the compound path of masculine discrepancy stress *and* anger, and we replicated this serial multiple mediator model across all three IPV outcomes.

### Supplemental Analyses

In supplemental analyses (not shown), we replicated our focal regression estimates with post-stratification weights. Because these results are substantively identical to the unweighted analyses reported in our tables, we retained our original analyses. We also failed to observe any “exposure-mediator” interactions (i.e., statistical interactions between our focal predictor variables and our mediator variables) (VanderWeele, 2015). Finally, we considered several additional background variables, including the presence of children under the age of 18, rural residence, region of residence, and religiosity. None of these variables had any substantive impact on our focal associations.

## Discussion

Although previous research has consistently shown that sexual dysfunction can be an outcome of IPV among women, few studies have considered whether sexual dysfunction might also lead some men to perpetrate IPV. In an effort to extend prior work, we tested whether men who experience sexual dysfunction are more likely to commit acts of IPV than other men. We also drew from GST and the concept of compensatory masculinity to test whether male sexual dysfunction increases the risk of IPV perpetration through the mechanisms of masculine discrepancy stress and anger.

Although we initially found that sexual dysfunction was associated with higher rates of any IPV perpetration, we determined that this association was driven by sexual IPV perpetration because sexual dysfunction was unrelated to physical IPV perpetration. The unique link between sexual dysfunction and sexual IPV perpetration is consistent with a process whereby some men “cope with perceived sexual difficulties through sexual coercion” (Carvalho et al., 2013, p. 1752). Some men who experience sexual dysfunction may employ (consciously or unconsciously) more direct acts of sexual IPV to project sexual power in a maladaptive effort to offset any perceived loss of virility and masculinity. Carvalho et al. (2013, p. 1746) also explain that “when individuals anticipate erectile difficulties, they may feel pressured to initiate sexual intercourse immediately after the perception of erection.” Both processes can help to explain the practice of demanding sex without condoms or other forms of sexual coercion. We note that our results support previous research on sexual dysfunction and sexual aggression against women among Portuguese college students (Carvalho et al., 2013). To our knowledge, ours is the first population-based study of American adults to document any association between male sexual dysfunction and IPV perpetration.

We observed that sexual dysfunction was associated with higher levels of masculine discrepancy stress and anger. Masculine discrepancy stress was also positively associated with anger. These patterns are consistent with the idea that male sexual dysfunction could pose a direct challenge to basic elements of the masculine role, especially those related to virility and sexual prowess (Fergus et al., 2002; Oliffe, 2005; Potts, 2000). We also confirm that threats to masculinity can be emotionally distressing for men (Berke et al., 2017; Chambers et al., 2017; Dahl et al., 2015; Eisler et al., 1988; Vescio et al., 2021).

Although masculine discrepancy stress was unrelated to IPV perpetration, our results demonstrated that men who reported higher levels of anger tended to report higher rates of any IPV perpetration and physical and sexual IPV perpetration. Our null findings for the association between masculine discrepancy stress and IPV perpetration failed to replicate previous studies based on small nonprobability samples of adolescents and men (Berke et al., 2017; Reidy et al., 2014, 2018). We note that Reidy and colleagues (2018) were also unable to observe any association between masculine discrepancy stress and physical IPV perpetration. The robust associations between anger and IPV perpetration support general conclusions of previous meta-analytic reviews (Birkley & Eckhardt, 2015; Norlander & Eckhardt, 2005).

Finally, our mediation analyses confirmed that sexual dysfunction was indirectly associated with all three IPV perpetration outcomes through the compound path of masculine discrepancy stress and anger. Although anger was a sufficient link between sexual dysfunction and IPV perpetration, masculine discrepancy stress was not. The IE of sexual dysfunction on IPV perpetration through masculine discrepancy stress was only observed through the lynchpin mechanism of anger. Empirically, our mediation model is noteworthy because it confirms social and emotional pathways through which male sexual dysfunction might increase the risk of IPV perpetration. Theoretically, our model is important in the sense that it offers a novel and direct test of GST (Agnew, 1992, 2001, 2007) and an indirect test of compensatory masculinity (Cheung & Chiu, 2021; DeMaris et al., 2003; Johnson & Ferraro, 2000). In future GST research, sexual dysfunction may be considered a strain related to the failure to achieve the positively-valued goals of health, virility, and masculinity among men (Agnew, 2007; Messerschmidt, 1993; Schroeder et al., 2011). More direct quantitative and qualitative assessments of the power and status motivations of perpetrators are needed to directly test the precise elements of compensatory masculinity (Cheung & Chiu, 2021; DeMaris et al., 2003; Johnson & Ferraro, 2000; Melzer, 2002; Pyke, 1996).

Although our study contributes to previous work, both theoretically and empirically, our work is limited in several respects. Because our analyses are based on a cross-sectional design, no causal or temporal inferences can be made. We suggest that sexual dysfunction might predict IPV perpetration; however, we acknowledge that a more rigorous analysis would use longitudinal data to assess changes in sexual dysfunction, masculine discrepancy stress, anger, and IPV. Because our IPV measures are limited to only a few items and dimensions, the veracity of our theoretical model is contingent upon replication and extension with more detailed assessments of IPV, including measures omitted from our analyses (e.g., psychological aggression and stalking). We also recognize that the frequency and severity of IPV can vary across studies and populations. Because our sample is characterized by a limited incidence of sexual IPV, we would like to see our model tested with larger samples and more established surveys like the *National Crime Victimization Survey* and the *National Intimate Partner and Sexual Violence Survey*. Finally, there is also the possibility of social desirability bias in self-reports of sexual dysfunction and IPV perpetration. We note that CHAPS employed self-administered surveys to minimize the potential for bias in reporting (e.g., the experience of shame from reporting ED or IPV to another person).

## Conclusion

With these limitations in mind, our mediation analyses suggest that male sexual dysfunction may increase the risk of IPV perpetration through the mechanisms of masculine discrepancy stress and anger. In addition to addressing our limitations, future work should consider alternative theoretical models. For example, GST research often incorporates conditioning factors to assess subgroup variations in the effects of strains and negative affective states on criminal behavior. Given a larger sample size, one could assess whether the links in our theoretical model vary according to theoretically relevant subgroups. One question is whether the association between sexual dysfunction and IPV perpetration varies across race and ethnic groups. Another question is whether the IE of sexual dysfunction on IPV perpetration through masculine discrepancy stress varies by socioeconomic status (i.e., moderated mediation). Research along these lines would provide a more thorough understanding of the link between sexual dysfunction and IPV perpetration.
